# A Study of Zn-Ca Nanocomposites and Their Antibacterial Properties

**DOI:** 10.3390/ijms23137258

**Published:** 2022-06-29

**Authors:** M. I. Torres-Ramos, U. J. Martín-Camacho, J. L. González, M. F. Yañez-Acosta, L. Becerra-Solano, Y. K. Gutiérrez-Mercado, M. Macias-Carballo, Claudia M. Gómez, O. A. González-Vargas, J. A. Rivera-Mayorga, Alejandro Pérez-Larios

**Affiliations:** 1 Laboratorio de Investigación en Nanomateriales, Agua y Energía, Departamento de Ingeniería, Centro Universitario de los Altos, Universidad de Guadalajara, Av. Rafael Casillas Aceves 1200, Tepatitlán de Morelos 47600, Mexico; isabel.torres7734@alumnos.udg.mx (M.I.T.-R.); ubaldo.martin2609@alumnos.udg.mx (U.J.M.-C.); 2 Especialidad en Odontopediatría, Centro Universitario de los Altos, Universidad de Guadalajara, Av. Rafael Casillas Aceves 1200, Tepatitlán de Morelos 47600, Mexico; juanluis_8@hotmail.com; 3 Laboratorio Biotecnológico de Investigación y Diagnostico, Departamento de Clínicas, División de Ciencias Biomedicas, Centro Universitario de los Altos, Universidad de Guadalajara, Av. Rafael Casillas Aceves 1200, Tepatitlán de Morelos 47600, Mexico; luis.becerra@cualtos.udg.mx (L.B.-S.); yanet.gutierrez@academicos.udg.mx (Y.K.G.-M.); monserrat.macias@cualtos.udg.mx (M.M.-C.); 4 Departamento de Química, División de Ciencias Naturales y Exactas, Campus Guanajuato de la Universidad de Guanajuato Noria Alta s/n, Col., Noria Alta, Guanajuato 36050, Mexico; claudia.martinez@ugto.mx; 5 Departamento de Ingeniería en Control y Automatización, ESIME-Zacatenco, Instituto Politécnica Nacional, UPALM, Av. Politécnico s/n, Col., Zacatenco, Alcadía Gustavo A. Madero, Ciudad de México 07738, Mexico; ogonzalez@ipn.mx; 6 Departamento de Química, Centro Universitario de Ciencias Exactas e Ingenierías, Universidad de Guadalajara, Boulevard Marcelino García Barragán, Calzada Olímpica, Guadalajara 44430, Mexico; joseriver88ku@hotmail.com

**Keywords:** nanoparticles, ZnO, antibacterial activity, nanocomposites, dental materials

## Abstract

This study aimed to develop Ca^2+^ doped ZnO nanoparticles (NPs) and investigate their antibacterial properties against microorganisms of dental interest. Zn-Ca NPs were synthesized by the sol-gel method with different concentrations of Ca^2+^ (1, 3, and 5 wt. %) and subsequently characterized by scanning electron microscopy (SEM), X-ray diffraction (XRD), UV-vis spectroscopy and Fourier transform infrared spectroscopy (FT-IR). The Kirby–Bauer method was used to measure antibacterial effects. NPs showed the wurzite phase of ZnO and bandgap energies (Eg) from 2.99 to 3.04 eV. SEM analysis showed an average particle size of 80 to 160 nm. The treatments that presented the best antibacterial activity were Zn-Ca 3% and Zn-Ca 5%. ZnO NPs represent an alternative to generate and improve materials with antibacterial capacity for dental applications.

## 1. Introduction

Nanotechnology is the most important dynamic exploration region in current of material science [1]. The significant growth in nanotechnology is best evidenced by the number of scientific articles and its many applications [2,3]. Recently, the scientific research community worldwide expressed interest in synthesizing metal and metal oxide nanoparticles (NPs) [4]. ZnO-NPs are of great importance due to their wide variety of applications in photocatalysis, water purification, and antibacterial disinfection. ZnO-NPs display properties that are distinct from those of typical NPs [5]. The biological activities of ZnO-NPs are size and morphology dependent, and are the subject of investigation by many researchers [3,6,7,8]. ZnO-NPs are considered a multi-purpose option and in recent years, research has focused on these metallic NPs due to their remarkable antimicrobial properties [9,10]. The antibacterial effects of these nanostructured agents is attributed to the high surface/volume ratio since it provides a greater contact area with agents in the environment. The ability to easily penetrate cell membranes disrupts various intracellular processes, resulting in high reactivity and antibacterial activity [11,12]. The incorporation of ZnO into dental components has received special attention, representing an effective alternative for biomedicine, specifically in oral health [13]. The antibacterial properties of ZnO as a nanocomposite can be used against Gram-negative bacteria, such as *Escherichia coli* (*E. coli*) [13,14], as well as Gram-positive bacteria, such as *Enterococcus faecalis* (*E. faecalis*) and *Staphyococcus aureus* (*S. aureus*), and it has gained interest for the elimination of these bacteria from the oral cavity [3,6,7].

Although antimicrobial compounds have been reported to decrease the occurrence of dental disease, the use of antibiotics and chemical bactericides can have a negative impact on the bacterial flora of the oral cavity and intestinal tract [15,16]. Since pathogens can acquire resistance against different antibiotics, agents that are characterized for having remarkable antibacterial activity and do not develop resistance are now in high demand [17,18]. Bacterial growth in the oral cavity is the main cause of secondary caries [19]. Improvement in the antibacterial properties of dental composites can effectively decrease the occurrence of secondary caries [20]. Presently, two methods are usually used to improve the conditions of the oral cavity. The first method involves the addition of antibacterial agents such as chlorhexidine (CHX), fluoride or silver ions into the resin composite whereas the second method involves the use of nanotechnology (NP’s, like ZnO) that provided an effective method for delivering a payload with antibacterial effects [2,21]. Several researchers have tried to improve the antibacterial properties of ZnO by doping it with other materials that have improved the inhibition of bacteria present in the oral cavity [22,23,24]. The use of Ca^2+^ has mainly been seen in bioactive glasses that have been synthesized by different methods [25,26] due to the need to improve the biocompatibility of materials.

Based on the above, this work aims to present the first report of a Zn-Ca nanocomposite applied in the dental field that was synthesized with different Ca^2+^ molar ratios. The NPs were labeled as Zn-CaX, where X represents the molar ratio of Ca^+^, and the antibacterial properties of each formulation were evaluated against strains of oral interest, such as *Staphylococcus aureus* (*S. aureus*), *Escherichia coli* (*E. coli*), *Enterococcus faecalis (E. faecalis)*, *Streptococcus mutans* (*S. mutans*), *Veillonella parvula* (*V. parvula*), *Fusobacterium nucleatum* (*F. nucleatum*), *Actinomyces odontolyticus* (*A. odontolyticus*).

## 2. Results

**Figure 1:**
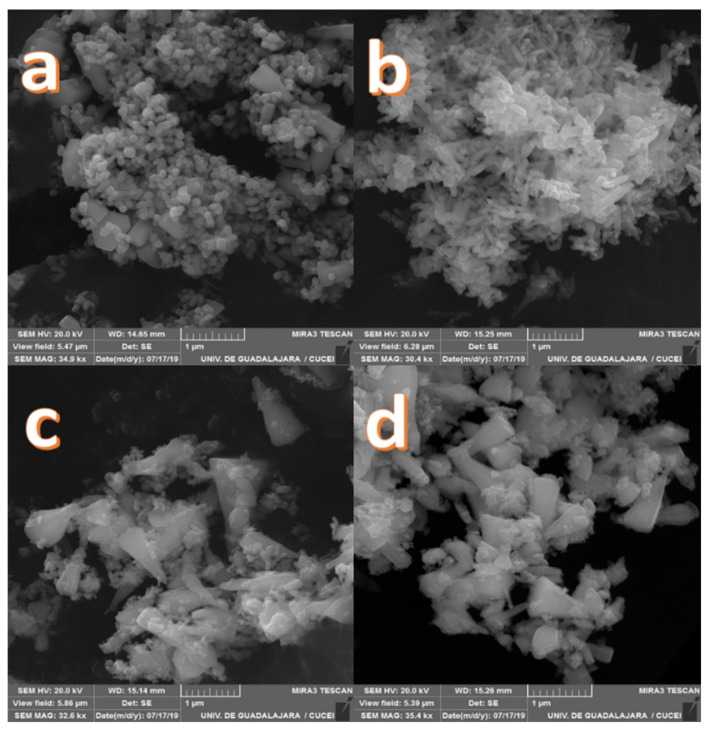
Scanning electron microscopy images of Zn-Ca nanocomposites. (**a**) ZnO, (**b**) Zn-Ca 1, (**c**) Zn-Ca 3, (**d**) Zn-Ca 5.

The images obtained by Scanning Electron Microscopy (SEM) of the Zn-Ca nanocomposites (Figure 1) show spherical and conical morphologies with a hexagonal base, and in some cases, rod-like morphologies were observed (Figure 1b–d), this was confirmed with Transmission Electron Microscopy (TEM). Table 1 shows the average particle sizes of each nanomaterial, as measured by ImageJ analysis software. XRD diffraction patterns of the Zn-Ca and ZnO nanocomposites showed characteristic peaks with an index Miller at (100), (002), (101), (102), (110), (103) y (112) (Figure 3). FT-IR spectroscopy (Figure 4) showed bands at 548, 692, 879, 2334, y 2366 cm−1. The UV-vis study (Figure 9) showed an optical absorption at 400 nm. Antibiograms (Figure 10) for Gram-negative bacteria (*E. coli*, *V. párvula* y *F. nucleatum*) showed inhibition halos less than 15 mm, with the best material being Zn-Ca5%, whereas the inhibition halos for Gram-positive bacteria (*E. faecalis*, *S. Aureus*, *S. mutans*, y *A. odontolyticus*) had an average size of 20 mm, and Zn-Ca 3% was the best material.

## 3. Discussion

The presented study was based on the implementation of a nanocomposite (Zn-Ca) as an antibacterial agent. The use of nanomaterials in the dental field has been reported [13,14,15,16,17] as an alternative to currently used treatments [4]. The implementation of calcium ions in nanocomposites provided the material with the specific characteristics demonstrated in this study. It is known that the structural morphology of zinc oxide nanoparticles encompasses spherical and hexagonal configurations [27], however, SEM microscopy allowed us to observe an increase in size as the percentage of doping increased [28]. This behavior is due to the ionic size of the Ca^2+^ ion, which is larger than the guest cation Zn^2+^ [29]. The substitution of Ca^2+^ with a larger radius in the Zn^2+^ sites resulted in an increase in the size of the nanoparticle, which agrees with other investigations where the concentration of the dopant directly influenced the morphology and size of the nanoparticles [30,31,32,33,34].

**Figure 2:**
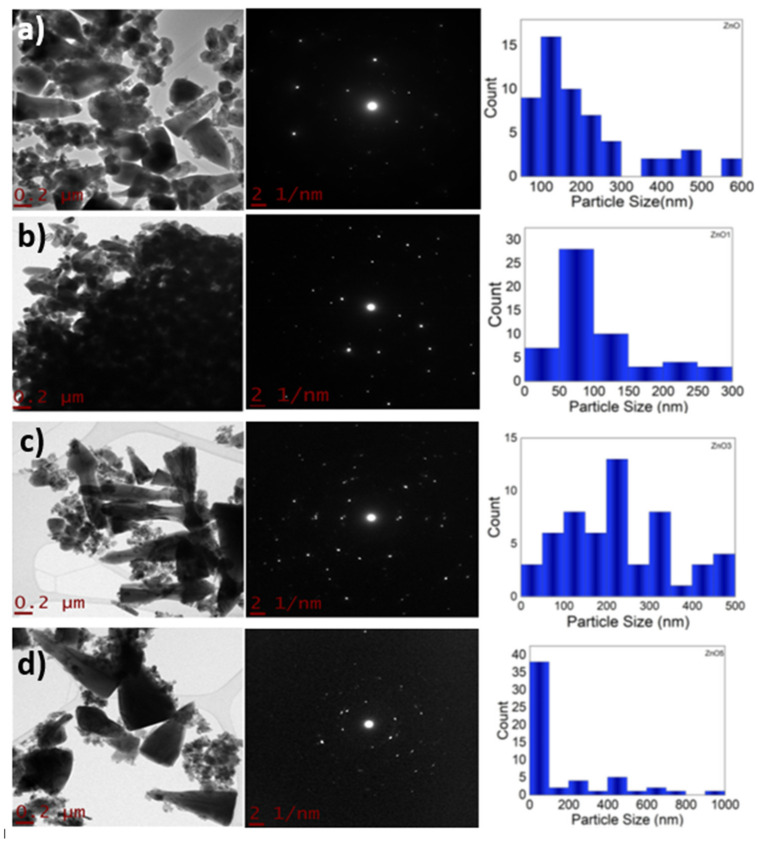
Transmission electron microscopy of Zn-Ca nanocomposites. (**a**) ZnO, (**b**) Zn-Ca 1, (**c**) Zn-Ca 3, (**d**) Zn-Ca 5.

In Figure 2, TEM images of ZnO shows spindle shaped nanoparticles [35]; however, as the Ca content increases, the size of Zn particles increases and the appearance of small particles corresponding to Ca was observed. To determine the size distribution of the nanoparticles, software was used measuring nanoparticle size at 50 nm [34].

**Figure 3:**
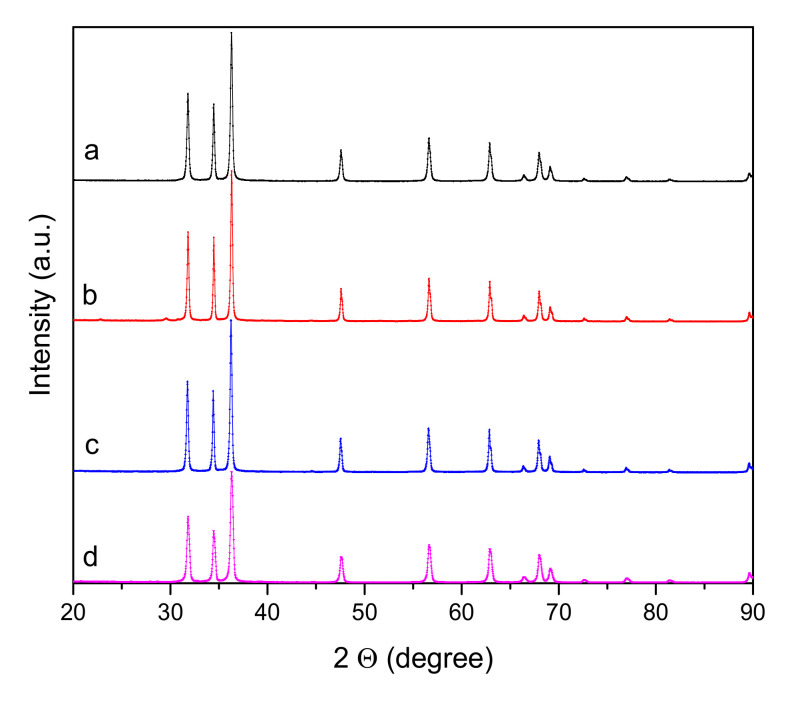
X-ray diffraction patterns of Zn-Ca nanocomposites. (**a**) ZnO, (**b**) Zn-Ca 1, (**c**) Zn-Ca 3, (**d**) Zn-Ca 5.

Regarding the crystallinity of the material, the XRD diffraction patterns (Figure 3) were found to be in agreement with the standard diffraction patterns for the wurtzite hexagonal phase for ZnO according to a crystallographic chart (JCPDS 01-079-0206) [36]. The diffraction peaks reveal the presence of ZnO but not Ca^2+^. These results are in agreement with reported studies that used the sol-gel method to synthesize the material, and the dopant was not detectable by XRD [37,38,39,40]. The average sizes of the ZnO and the Zn-Ca nanocomposites were calculated by the Scherrer equation (Table 1) and agree with those reported in ZnO studies [30,41]. The crystal size increased in proportion with the increase in the concentration of the dopant, except for the Zn-Ca5 material. This was due to saturation by the dopant, which generates a decrease in crystal size [31]. The lattice parameters were calculated using the Bragg’s law equation, obtaining similar values for the parameters between the nanocomposites, which indicates that the incorporation of calcium ions does not modify the morphology and maintains the phase of the material [42].

**Figure 4:**
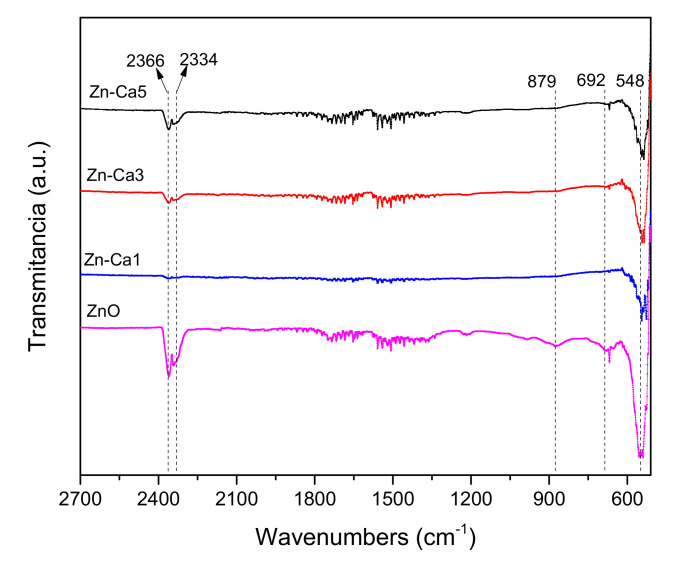
FT−IR spectra of Zn-Ca nanocomposites.

**Figure 5:**
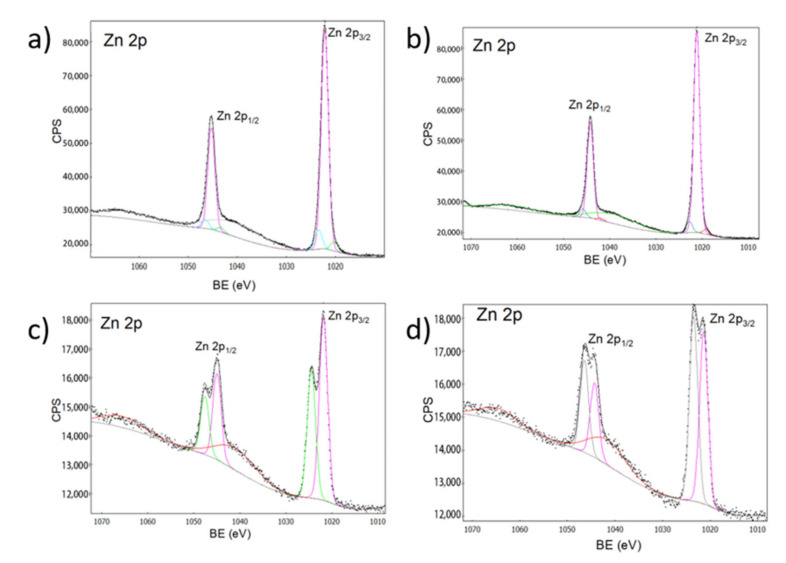
XPS spectra for zinc. (**a**) ZnO, (**b**) Zn-Ca 1, (**c**) Zn-Ca 3, (**d**) Zn-Ca 5.

FT-IR spectroscopy (Figure 4) confirmed the wurzite phase of the material, identifying the presence of tetrahedral groups of ZnO and the Zn-O-Ca bond that make up this structure [43]. Characteristic bands associated with functional groups present in ZnO were also detected, which correspond to the metal-oxygen vibration modes, shows bands at 548 and 692 cm^−1^ corresponding to vibrational modes of Zn-O and Zn-OH [44]. The absorption peaks at 2334 and 2366 cm^−1^ were assigned to absorption levels that reveal the presence of C-H stretching vibrations due to the precursor used in the synthesis [36]. This analysis, together with the XPS (Figure 5) confirm the presence of Zn in the nanocomposite.

An XPS analysis was performed for the samples, obtaining signals corresponding to zinc (Zn 2p), calcium (Ca 2p), oxygen (O 1s) and carbon (C 1s). In Figure 5, we observed the characteristic peaks for Zn 2p_1/2_ and Zn 2p_3/2_ [33]. The signals observed around 1047 and 1024 eV were assigned to Zn in octahedral sites, and the increase in the intensity of these signals with the increase in doping is appreciable, which is related to the increase in the concentration of antisite defects [45,46].

**Figure 6:**
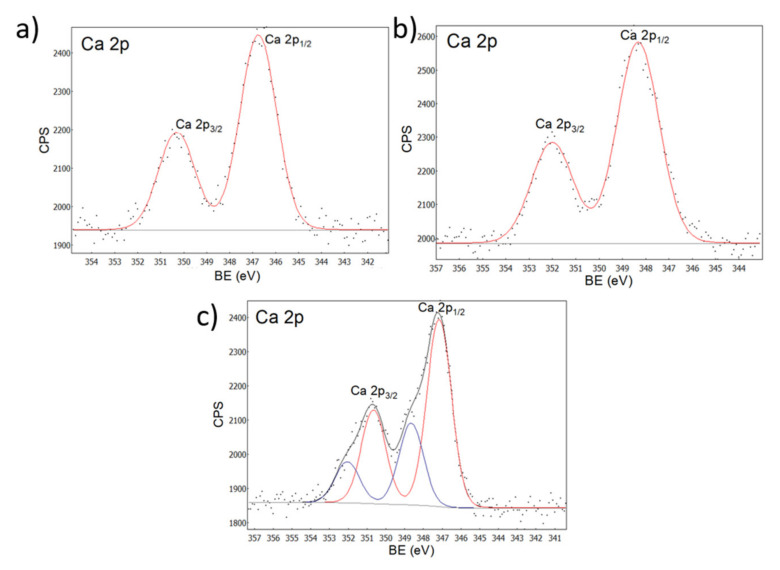
XPS spectra for calcium. (**a**) Zn-Ca 1, (**b**) Zn-Ca 3, (**c**) Zn-Ca 5.

The calcium spectra (Figure 6) showed peaks belonging to Ca 2p_3/2_ at 351 eV and Ca 2p_1/2_ at 347 eV, and the deconvolution of the spectrum in Figure 5c suggests interactions between COO- and Ca^2+^ in accordance with reports by other authors [47,48].

**Figure 7:**
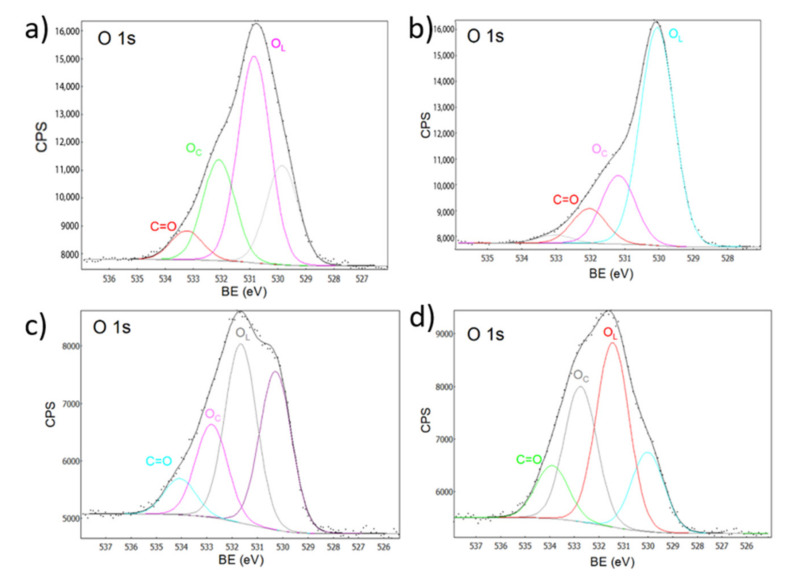
XPS spectra for oxygen. (**a**) ZnO, (**b**) Zn-Ca 1, (**c**) Zn-Ca 3, (**d**) Zn-Ca 5.

Figure 7 shows the XPS analysis corresponding to oxygen. The peak corresponding to O 1s can be broken down into various Gaussian components, where the band present at approximately 533 eV belonged to chemisorbed oxygen (O_C_), the band at 531 eV belonged to lattice oxygen (O_L_) species, whereas the signals present at approximately 534 eV can be assigned to the C = O bond in the nanocomposites, where increases in intensity were proportional to the percentage of the dopant [33,49].

**Figure 8:**
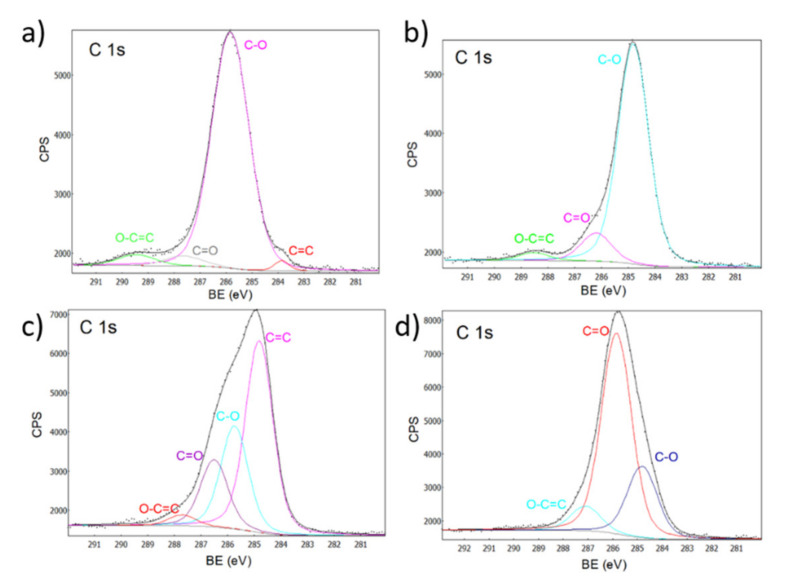
XPS spectra for carbon. (**a**) ZnO, (**b**) Zn-Ca 1, (**c**) Zn-Ca 3 and (**d**) Zn-Ca 5.

The spectra for carbon (C 1s) is shown in Figure 8, where the peak deconvolutions at 289, 287, 285, and 284 correspond to O-C = C, C = O, C-O and C = C, respectively [48]. The spectra indicates a shift towards higher binding energies when calcium ions are used as a dopant [33,49].

**Figure 9:**
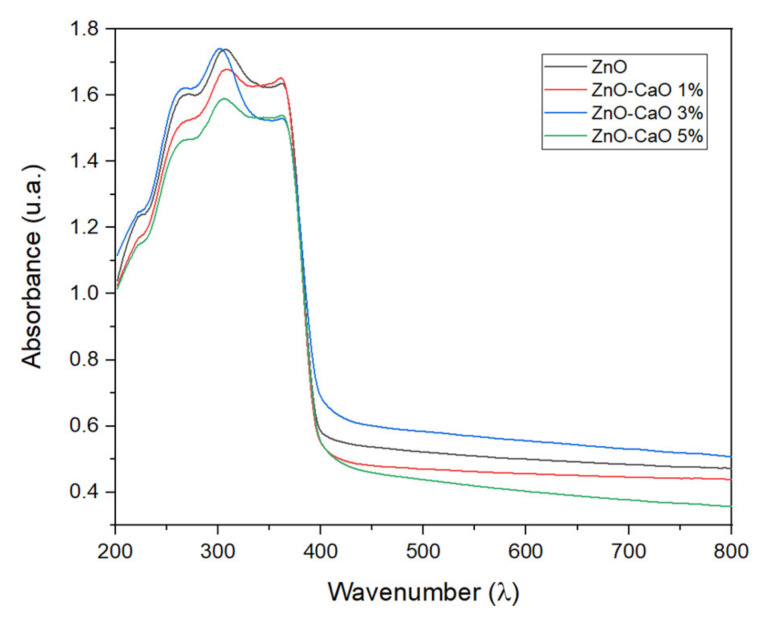
UV-Vis spectra and determination of the bandgap energies for ZnO, Zn-Ca 1, Zn-Ca 3, and Zn-Ca 5.

The optical absorbances obtained in the UV-vis analysis (Figure 9) can be attributed to the Zn-O electron transitions of ZnO. The results show a small shift in the red region (3.3 a 2.99 eV) [50]. Thus, the incorporation of CaO into ZnO produces only small variations in the band gap energy, Eg. Adding donor or acceptor impurities to a semiconductor creates energy levels near the conduction or valence band edges, as seen for the Zn-Ca 1 and Zn-Ca 5 nanocomposites. This behavior is in agreement with that reported by various authors [31,32,42], and indicates that the incorporation of calcium ions to the material presents small variations in the energy of the forbidden band without generating modifications in the stability of ZnO.

**Figure 10:**
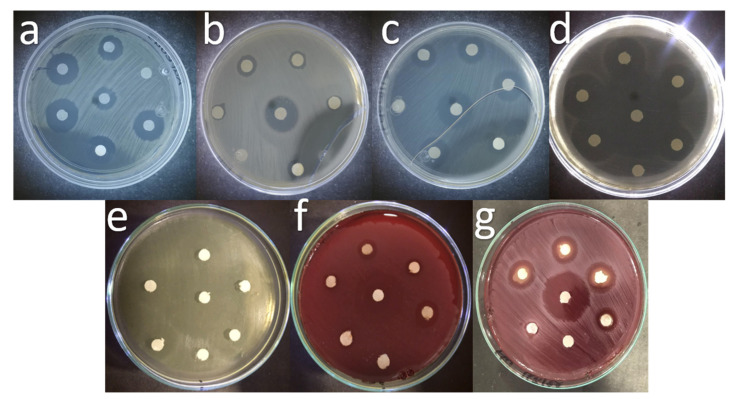
Antibiograms of the evaluated treatments, (**a**) *E. coli*, (**b**) *E. faecalis*, (**c**) *S. Aureus*, (**d**) *S. mutans*, (**e**) *A. odontolyticus*, (**f**) *V. parvula*, (**g**) *F. nucleatum.*

The antibacterial properties of the evaluated nanocomposite can be attributed to reactive oxygen species (ROS) [51,52] that inhibit bacterial growth because ZnO NPs release Zn^2+^ ions that cross the cell wall and react with cytoplasmic content. It is also known that ZnO NPs produce H_2_O_2_, which is a strong oxidant capable of causing great damage to the cell membranes of bacteria [50,53]. These results are in accordance with Kim et al. who evaluated the antimicrobial activity of films containing ZnO and determined that as the concentration increased, the antimicrobial activity of the film produced a better inhibition halo (Figure 10) [54]. In addition, the antimicrobial activity of ZnO and its nanocomposites synthesized in this work showed better activity than previous reports where the inhibition halos ranged from 12–15 mm [55,56] Table 2.

## 4. Materials and Methods

### 4.1. Chemical Reagents

Zinc oxide (ZnO) was obtained from zinc acetate dihydrate (C_4_H_6_O_4_Zn * 2H_2_O, Sigma Aldrich, St. Louis, MO, EE. UU.), calcium ions were obtained from calcium nitrate A.C.S. (CaN_2_O_6_ * 4H_2_O, MEYER, CDMX, MX.).

### 4.2. Nanomaterial Synthesis

Zn-CaX nanoparticles were synthesized by the sol-gel method with some modifications by using zinc acetate as a precursor [38] and Ca^2+^ as the dopant. For this purpose, 14 g of Zn acetate was dissolved in 140 mL of ethanol (CTR, VA, EE. UU.) in a three-mouth flask with the following amounts of Ca^2+^: 1% (Zn-Ca 1), 3% (Zn-Ca 3), and 5% (Zn-Ca 5). Then, a few drops of HNO_3_ (1 M, Sigma Aldrich) were added to adjust the pH of the solutions to 3. Each solution was heated under reflux at 80 °C for four hours with magnetic stirring. The solution was then cooled down to 0 °C for 18 h. The resulting gel was dried at 100 °C and annealed at 500 °C for 4 h in a static air atmosphere (heating rate of 2 °C min^−1^). A similar procedure was followed for the synthesis of ZnO nanoparticles.

### 4.3. Sample Characterization

The morphology of the materials was observed by scanning electron microscopy (Tescan, MIRA 3LMU, LDN, UK) operated at 20 kV. High-resolution images were acquired using a high-resolution transmission electron microscope (Jeol microscope, JEM-ARM200F, Boston, MA, USA.) operated at 200 kV. The resulting images were analyzed using Gatan Micrograph software v. 3.7.0 (Pleasanton, CA, USA).

The absorption spectra of the materials were acquired by a UV-Vis DRS (Shimadzu UV-2600, Tokyo, Japan) provided with an integration sphere suitable for diffuse reflectance studies. The UV-Vis DRS spectra were obtained from 190 to 900 nm wavelength. From the plot, the bandgap energy was calculated using Plank’s equation [32] as follows:

(1) $$Eg=\frac{1239.8}{\lambda }$$

where energy (*E_g_*) = band gap energy (eV), and wavelength (*λ*) = absorption peak value.

The X-ray powder diffraction patterns were acquired using an XRD Panalytical diffractometer (Empyrean, Almelo, Netherland) equipped with Cu Kα radiation (*λ* = 0.154 nm). Data were collected from 10° to 90° (2θ) with a scan rate of 0.02°/0.2 s. The average crystal size was determined using the Scherrer equation as follows:

(2) $$D=\frac{k\lambda }{\beta \cos\theta }$$

where *D* is the crystal size, *k* is the form factor (0.89), *λ* is the wavelength of Cu Kα radiation, *β* is the width evaluated at mid-high of the most intense diffraction peak and *θ* is the Bragg angle. The inter-planar distance (*d*) can also be evaluated from Bragg’s law as follows:

(3) 2dsinθ=nλ

The FT-IR spectra for the material were recorded with an FT-IR (Shimadzu, IRTracer-100, Tokyo, Japan) spectrophotometer using attenuated total reflectance (ATR) with a diamond waveguide (XR model). A detector with fast recovery deuterated triglycine sulfate (DTGS) (standard) was used for the analysis. The spectra were recorded at room temperature with 24 scans and 4 cm^−1^ of resolution from 4000 cm^−1^ to 400 cm^−1^. 

The interactions between chitosan and magnetite in the ChM composite and ChM-arsenic were characterized by X-ray photoelectron spectroscopy (XPS) using an XPS SPECS system (Berlin, Germany), which contains a Phoibos 150 analyzer and a 1D DLD detector. The XPS spectra were obtained with a monochromatic Al Kα source (1486.7 eV) working at 250 W (12.5 kV and 20 mA) and a base pressure of 3 × 10^−9^ mbar in the analytical chamber. The high-resolution scans were conducted with a pass energy of 15 eV and step sizes of 0.1 eV using a flood gun source with 20 µA of emission and 2 eV energy to compensate. Data were analyzed with Analyzer 2.21 software using Lorentzian–Gaussian curves after background subtraction [57].

### 4.4. Antibacterial Activity

Zn-Ca NPs were evaluated against *S. aureus* (ATCC 33862), *E. coli* (ATCC 8739), *E. Faecalis* (ATCC 19433), *S. mutans* (ATCC 25175), *V. parvula* (ATCC 10790), *F. nucleatum* (ATCC 25586) and *A. odontolyticus* (ATCC 17929) by using the disk diffusion method. Bacteria were inoculated 10^8^ CFU/mL onto Muller Hinton agar medium in petri dishes. Consequently a 6 mm diameter paper disc was placed on the test organism impregnated with nanoparticles (100, 200, 300, 400, and 500 μg/mL), prepared in sterile bi-distilled water. The plates were incubated at 37 °C for 24 h. Discs with ampicillin (10 μg/mL) were used as the positive control (C+), and discs impregnated with sterile bi-distilled water served as the negative control (C−). Antimicrobial activity was determined by measuring the zone of inhibition (mm) around the disc [41].

## 5. Conclusions

The results of this study show that ZnO synthesized with the sol-gel method does not present changes in its textural and surface properties, maintaining the characteristic wurzite phase of ZnO, as well as its morphology. The Zn-Ca nanocomposite had better antibacterial activity against Gram-negative bacteria, with an increase in efficacy proportional to the percentage of Ca. Zn-Ca nanocomposites represents an active line of research for the dental field, and the evidence suggests continuing the evaluation of the applicability of this nanomaterial and its interaction with other microorganisms of dental interest.

Researchers who are interested in carrying out similar research work are recommended to carry out a nitrogen physisorption analysis to determine the surface area of the material, cytotoxicity and cell viability studies, in addition to carrying out release profiles to determine the optimal doses of use.

## Figures and Tables

**Table 1:** Surface analysis of nanocomposites.

Material	Eg (eV)	Lattice Parameters		Crystallite Size (nm)
		a = b (Å)	c (Å)	
ZnO	3.03	3.2477	5.2035	42.13
Zn-Ca 1	2.99	3.2476	5.2024	47.93
Zn-Ca 3	3.04	3.2511	5.2059	52.82
Zn-Ca 5	3.01	3.2482	5.2041	34.61

**Table 2:** Antimicrobial activity of ZnO-CaO samples.

*Treatment*		*Concentration (μg/mL)*				
	Control (+)	100	200	300	400	500
*E. coli ATCC 8739*						
*ZnO*	19.3	6.33	7.33	7.33	7.67	8.67
*Zn-Ca 1%*	18.3	6.63	6.67	6.67	7.33	8.33
*Zn-Ca 3%*	18.6	8.33	8.67	11.33	12	13.67
*Zn-Ca 5%*	17.5	10	10	13	13.33	14.67
*E. faecalis ATCC 19433*						
*ZnO*	23.6	8.33	10	10.67	14.33	15.33
*Zn-Ca 1%*	22.6	6.33	9	9	9.33	10.67
*Zn-Ca 3%*	23.5	11	12.33	13.7	16.33	17.67
*Zn-Ca 5%*	22	11.33	14	15.67	16.33	18.67
*S. Aureus ATCC 33862*						
*ZnO*	23.8	13.5	14.3	15	16.3	17.6
*Zn-Ca 1%*	25	15	15.3	17	19.5	20
*Zn-Ca 3%*	25.3	19	19.6	23	24.5	31.5
*Zn-Ca 5%*	22.4	20	20.6	22.6	25	25.6
*S. mutans ATCC 25175*						
*ZnO*	25.5	14.5	17	18	19.5	20.5
*Zn-Ca 1%*	25	15.5	16	18	19.5	20
*Zn-Ca 3%*	25.5	19	21	22.5	23.5	24.5
*Zn-Ca 5%*	26.5	16.5	17	19.5	21	23.5
*A. odontolyticus ATCC 17929*						
*ZnO*	26.4	14.5	17.5	20	22	24.5
*Zn-Ca 1%*	25.6	15	18	24	25	27
*Zn-Ca 3%*	26	25	28	30	31	31.3
*Zn-Ca 5%*	27.3	20	27	30	32	35
*V. párvula ATCC 10790*						
*ZnO*	17.3	9.2	9.6	10.3	10.9	11.4
*Zn-Ca 1%*	17.9	10.3	10.6	10.8	11.3	11.6
*Zn-Ca 3%*	17	11.4	11.9	12.5	12.7	12.9
*Zn-Ca 5%*	18.2	12.3	12.8	13.0	13.4	13.7
*F. nucleatum ATCC 25586*						
*ZnO*	17.6	10.3	10.7	11.2	11.8	12.3
*Zn-Ca 1%*	18.4	10.5	10.9	11.6	12.0	12.7
*Zn-Ca 3%*	18	12.6	12.95	13.4	13.7	14.1
*Zn-Ca 5%*	17.3	13.3	13.8	14.2	14.6	14.9

## Data Availability

Not applicable.
